# A microblog content credibility evaluation model based on collaborative key points

**DOI:** 10.1038/s41598-022-19444-6

**Published:** 2022-09-08

**Authors:** Ling Xing, Jinglong Yao, Honghai Wu, Huahong Ma

**Affiliations:** College of Information Engineering, Henan University of Science and Technology, Luoyang, 471023 Henan China

**Keywords:** Computer science, Information technology

## Abstract

The spread of false content on microblogging platforms has created information security threats for users and platforms alike. The confusion caused by false content complicates feature selection during credibility evaluation. To solve this problem, a collaborative key point-based content credibility evaluation model, CECKP, is proposed in this paper. The model obtains the key points of the microblog text from the word level to the sentence level, then evaluates the credibility according to the semantics of the key points. In addition, a rumor lexicon constructed collaboratively during word-level coding strengthens the semantics of related words and solves the feature selection problem when using deep learning methods for content credibility evaluation. Experimental results show that, compared with the Att-BiLSTM model, the F1 score of the proposed model increases by 3.83% and 3.8% when the evaluation results are true and false respectively. The proposed model accordingly improves the performance of content credibility evaluation based on optimized feature selection.

## Introduction

Online social network is a new mean of obtaining information from people. Due to the large volume of user-generated content, researchers use various techniques, such as content credibility evaluation or data mining to evaluate this information automatically^1–4^. Microblog is one of the important platforms in onilne social networks. The development of microblogging has greatly accelerated the depth and speed of information exchange between users^5^. However, while microblogging improves convenience for users, it also reduces the cost of disseminating false content. The dissemination of false content hurts social stability, disrupts people’s normal lives and endangers network information security^6,7^. It is therefore important to evaluate the credibility of microblog content, a practice that has numerous benefits^8,9^. Related deep learning methods have strong feature learning capabilities and can learn deep features from microblogs to achieve better credibility evaluation results^10^.

Microblog content is created and disseminated at specific times and via specific channels, which complicates research into deep learning-based content credibility evaluation^11^. The concept of content semantics began to be put forward, and the content semantics after mining are called features^12–15^. False content is highly confusing, meaning that analyzing and mining the characteristics of false content can produce a better evaluation effect. Therefore, feature selection is of great importance to content credibility evaluation^16^. To solve the feature selection problem in the content credibility evaluation context, researchers have proposed that deep learning be used to mine microblog text features^17^. Geng et al.^18^ use the attention mechanism to obtain the features that are most useful for the task in question. Kumar et al.^19^ developed a multi-head attention mechanism to obtain sentence-level key features. The multi-head attention model has achieved outstanding performance in mining multiple key point features^20^. Sangeetha et al. employ a multi-head attention mechanism to process sentence input sequences in parallel^21^. Khan et al. introduced a multi-head attention mechanism in a convolutional neural network to ensure that the model automatically selects key features^22^.

The acquisition of microblog features begins at the word level and moves to the sentence level. The introduction of the lexicon can add more task-related word information, leading to stronger semantics^23,24^. However, no lexicon designed for content credibility evaluation has yet been developed.

Based on the above analysis, this paper proposes a microblog content credibility evaluation model based on collaborative key points. The main innovations of our work can be summarized as follows: (1) using fake microblogs to extract the basic rumor word set, then employing an iterative algorithm based on the Word2Vec word vector cosine similarity calculation method to expand the rumor word database; (2) using the improved TF-IDF algorithm to calculate the comprehensive rumor value of the words in the rumor lexicon, after which the comprehensive rumor value of the words and the words themselves are vectorized, such that the semantics are strengthened to a degree that aids in the acquisition of microblog key points; (3) employing the multi-head attention mechanism twice—at the word level and sentence level—to obtain the microblog key points, which improves the text self-attention performance and enhances the model’s ability to evaluate the credibility of the content.

The rest of this paper is structured as follows. “Related works” section introduces the related works of content credibility evaluation based on deep learning. Section “Proposed credibility evaluation model” section describes the model proposed in this paper. “Experimental results and evaluation” section discusses our experiments. And “Conclusion” section concludes.

## Related works

As microblogging has continued to develop, information security issues caused by false information have attracted the attention of researchers. To date, researchers have proposed many methods to solve the feature selection problem in content credibility evaluation.

Common methods of this kind are mainly based on deep learning^25^. Unlike classifier-based methods^26,27^, deep learning methods can mine deep features of the content. Ma et al.^28^ used Recurrent Neural Networks (RNNs) to learn the features of the content. Duong et al.^29^ used RNN to combine the text and text source characteristics, while Chen et al.^30^ used RNN to learn the features in text and comments. It can be observed from these works that the introduction of other effective features in addition to the text boosts the performance of these methods. Torshizi et al.^31^ clustered the data, then used a long short-term memory network (LSTM) to analyze each cluster and determine whether the content is truthful. To solve the context information acquisition problem, an improved bidirectional long short-term memory network (BiLSTM) method is proposed^32,33^. Guo et al.^34^ used BiLSTM to process data in two directions and obtained text context information. Recently, substantial work has shown that pre-trained models on the large corpus can learn universal language representations, which are beneficial for content credibility evaluation tasks and can avoid training a new model from scratch^35^. And various pre-training tasks are proposed for different purposes, such as GloVe^36^ and BERT^37^.

When performing collaborative deep learning tasks, establishing or expanding a lexicon based on the characteristics of the task can help neural networks to more efficiently learn relevant information features. Wang et al.^38^ proposed an emotional vocabulary expansion method based on word spacing and mutual point information. Jia et al.^39^ added new contents and emotional symbols to the HowNet Emotion Dictionary to analyze the evolution of public opinion. Wang et al.^40^ used an improved dictionary classification method to calculate and label the emotional score of the content in the dataset and achieve emotional classification for microblogs. Zhang et al.^41^ used the TF-IDF algorithm to extract keywords from comments and construct an emotion dictionary based on word similarity. However, due to the wide variety of false information contained in microblogs, it is not possible to expand the thesaurus by mining the emotional information of certain words in a similar way to the sentiment classification task^42^, resulting in the current absence of a content credibility-related lexicon.

Subsequently, the researchers found that introducing the attention mechanism into the model can effectively improve the performance of the model. The attention mechanism processes large amounts of information and selects the information that is more critical to the goal. Xu et al.^43^ introduced the content focus mechanism to aggregate keywords in original tweets. Ghanem et al.^44^ focused on the emotional features generated by data to identify false information. Wu et al.^45^ constructed a propagation graph based on the propagation characteristics of false information and dynamically adjusted the weight of nodes in the graph using the attention mechanism. Fang et al.^46^ combined the multi-head attention model with Convolutional Neural Networks (CNNs) to select words that are more conducive to classification between levels, thereby achieving fake news detection.

## Proposed credibility evaluation model

**Figure 1:**
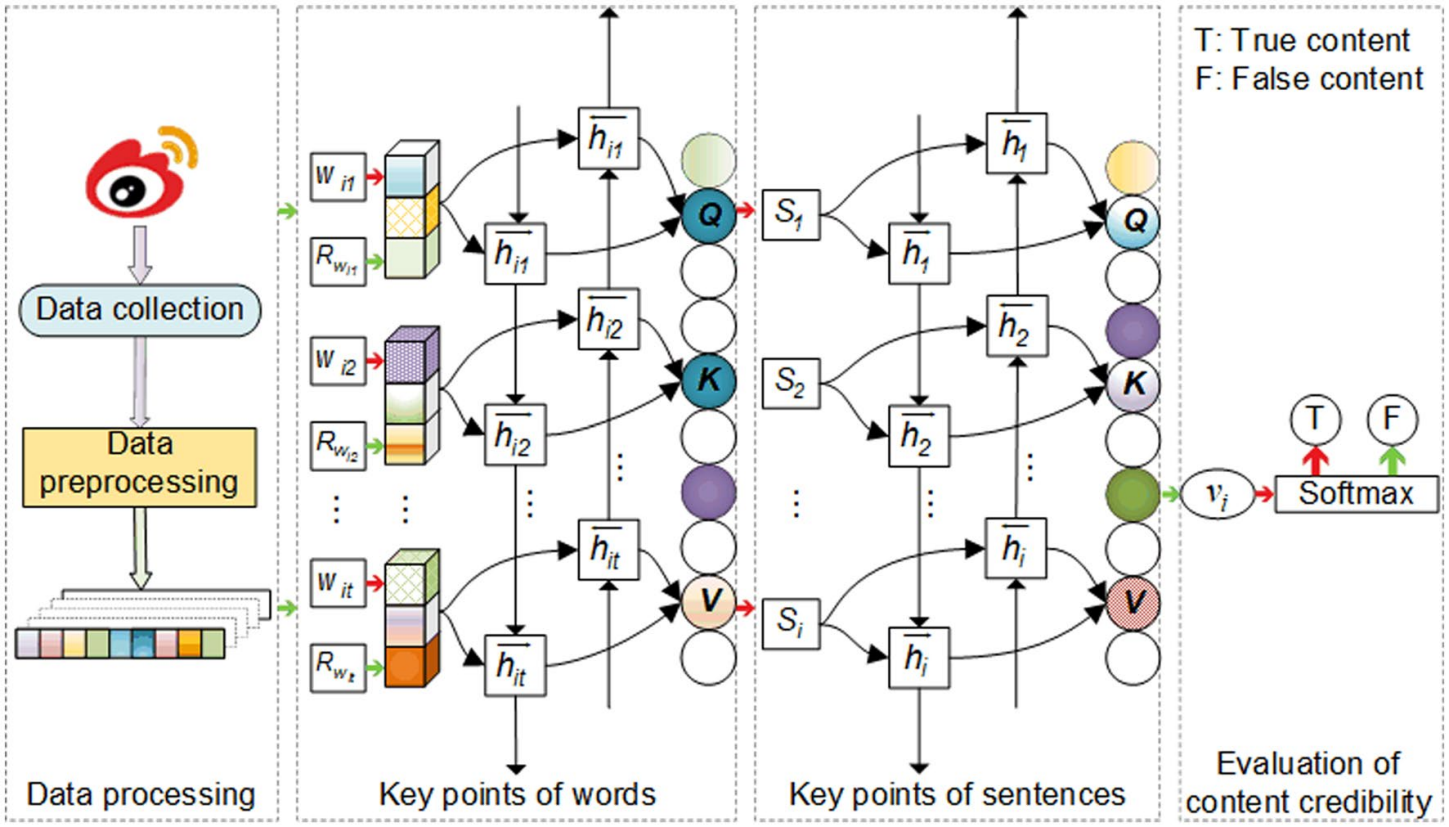
Microblog content credibility evaluation model based on collaborative key points.

Hierarchical attention networks (HANs) encode from the word level to the sentence level, which is an effective means of obtaining key points^47^. This paper uses a multi-head attention mechanism based on HAN; at the same time, it introduces a rumor lexicon to facilitate word coding, and accordingly builds a microblog content credibility evaluation model based on collaborative key points (CECKP). The overall structure of the model is illustrated in Fig. 1. This model is divided into four parts: data processing, the key points of words, the key points of sentences, and content credibility evaluation.

### Data processing

Due to the dearth of large, open and complete datasets appropriate for the present task, most relevant studies use the application program interface (API) provided by the platform to obtain data for their experiments^48^. In this paper, 15,000 points of false information and 15,000 of true information were extracted, from which an experimental dataset for content credibility assessment based on Sina Weibo (named the CECKP-Dataset) was constructed. The false information is derived from the results announcement section of the false information report in the Sina Microblog Community Management Center, while the true information is made up of verified content posted by well-known official accounts. In the subsequent word vector representation, it is necessary to convert the words and the comprehensive rumor value of these words into a vector form. It is accordingly necessary to construct a rumor vocabulary based on the characteristics of the words in microblogs containing false information. The remainder of this section will explain the construction of the rumor lexicon, its acquisition, its expansion, and the calculation of the comprehensive rumor value.

**Figure 2:**
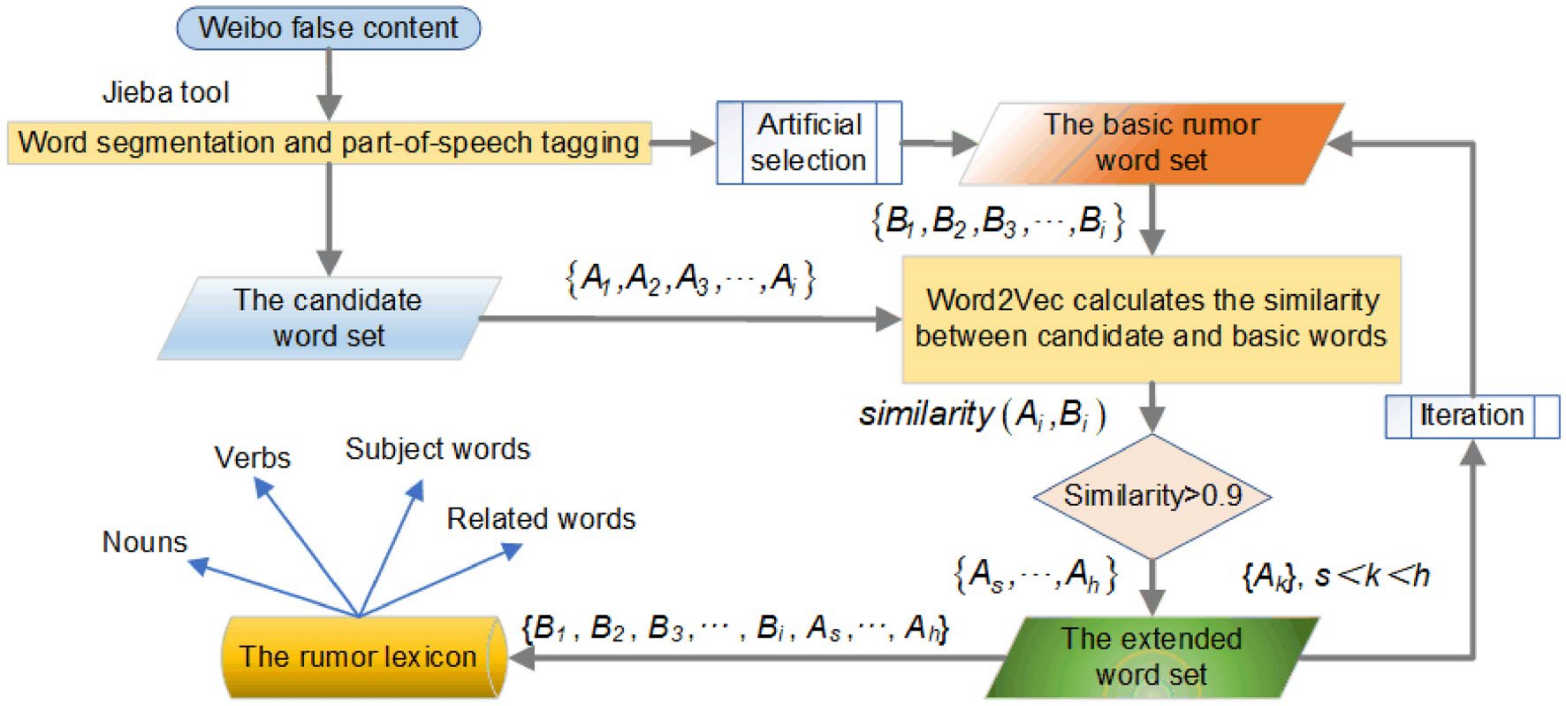
Flowchart of microblog rumor word database construction.

*Construction of the rumor lexicon* This paper sorts the false content published by the Sina Weibo community management center and uses a combination of manual screening and automatic expansion to construct a microblog rumor lexicon. The preprocessed lexicon is manually screened to obtain the basic rumor word set {*B*_1_,*B*_2_,*B*_3_,*…*,*B*_*i*_}. The remaining part is used as the candidate lexicon word set {*A*_1_,*A*_2_,*A*_3_,*…*,*A*_*i*_}, after which the Word2vec word vector cosine similarity calculation method is employed to calculate the similarity between the candidate words and the basic words^49^. Words that meet the initial requirements will enter the extended lexicon, after which some words in the extended lexicon will be put into the basic rumor lexicon again for iterative calculations until no new words can be obtained. The construction process is illustrated in Fig. 2.

*Acquisition of basic rumor word set*: First, duplicate content removal is carried out on the microblog false information corpus; here, we delete "@", "&", and other special symbols that cause interference. The added dictionary function of the Jieba word segmentation tool is then employed to integrate the Chinese word segmentation dictionary as an added dictionary used to mark the word segmentation and parts of speech on the microblog corpus. The dictionary integrates the Baidu and Sogou word banks, as well as some names of individuals and popular new terms. True and false content differs minimally in terms of structure and thus needs to be considered in combination with the actual situation. Nouns are generally the subject or indicator of the main meaning, and are thus better able to represent false information; moreover, “#” or “[]” are often used to mark key nouns and thus highlight the theme of the post. The application scenarios of verbs are limited by content and often appear in some false information. Related words refer to words that appear together in the text: when these words appear together, the text is more likely to be false. In this paper, nouns, verbs, subject words and related words are selected to construct a rumor lexicon.

*Expansion of the rumor lexicon*: To efficiently filter the candidate words in the corpus, the Word2Vec word vector cosine similarity calculation function is employed to calculate the similarity between each word in the candidate vocabulary set and the basic rumor word set. The similarity calculation formula is as shown in Eq. (1):

1 $$similarity\left( {A,B} \right) = \frac{{{\varvec{A}} \cdot {\varvec{B}}}}{{\left\| {\varvec{A}} \right\|\left\| {\varvec{B}} \right\|}} = \frac{{\sum\limits_{i = 1}^{n} {A_{i} \times B_{i} } }}{{\sqrt {\sum\limits_{i = 1}^{n} {\left( {A_{i} } \right)^{2} } } \times \sqrt {\sum\limits_{i = 1}^{n} {\left( {B_{i} } \right)^{2} } } }}$$

Here, ***A*** and ***B*** represent the word vector in the candidate word set and the basic rumor word set, respectively. If the similarity exceeds 0.9, the word is added to the extended lexicon {*A*_*s*_,*…*,*A*_*h*_}; the manually filtered extension {*A*_*k*_} is then added to the basic rumor word set, where *s* < *k* < *h*, after which incremental iterations are performed to mine more related words. When the algorithm cannot find new words, the iterations stop, and we can obtain the final rumor lexicon {*B*_1_,*B*_2_,*B*_3_,*…*,*B*_*i*_*,A*_*s*_*,…,A*_*h*_}. The relevant information regarding the constructed rumor lexicon is presented in Table 1.

**Table 1:** Information about the rumor lexicon.

Category	Number	Example
Noun	1687	Health, disaster, elderly
Verb	756	Crash, lead to, provocation
Subject	354	Blast, vacation, plastic
Connective	49	Child…abducted…, forward…free…

*Calculation of comprehensive rumor value*: In this paper, the improved TF-IDF algorithm is used to calculate the importance of the words in the rumor word database; these calculated results are then used as the comprehensive rumor value of the words in question. Because only part of the noun is modified in the false information, the requirements for the text frequency are relatively high, while the requirements for the inverse text frequency are relatively low. Accordingly, adjusting the weight in the formula makes TF more powerful than IDF. At the same time, to eliminate the influence of different microblog text lengths on the weight of words, the calculation formula is subjected to cosine normalization, with the word frequency taken as a logarithm to eliminate the influence of different word frequencies on the overall calculation.

The calculation formula of the comprehensive rumor value *R* is presented in Eq. (2):

2 $$R\left( w \right) = \frac{{\log_{2} \left( {\varphi tf + 1} \right) \times \log_{2} \left( {\frac{N}{{\left( {1 - \varphi } \right)idf}}} \right)}}{{\sqrt {\sum\limits_{i}^{{}} {\left( {\log_{2} \left( {\varphi tf_{i} + 1} \right) \times \log_{2} \left( {\frac{N}{{\left( {1 - \varphi } \right)idf_{i} }}} \right)} \right)^{2} } } }}$$

Here, *N* represents the total number of fake microblogs and *φ* is the weight of TF. The calculated comprehensive rumor value of the words in the fake microblog will be transformed into vectors and words, which are entered simultaneously into the model for training.

### Key points of words

The acquisition of word key points comprises three steps: word vector representation, word sequence coding, and word attention.

*Word vector representation*: The quality of word vector expression has an important influence on both the semantic expression of microblog texts and the effectiveness of credibility evaluation tasks. This paper introduces the constructed rumor lexicon into the word vector representation layer. The input word vector comprises two key parts: the word vector and the comprehensive rumor value of the word. The calculation formula is as shown in Eq. (3):

3 $${\varvec{x}}_{it} = {\varvec{W}}_{e} w_{it} \oplus R_{{w_{it} }} ,t \in \left[ {1,T} \right]$$

Here, ***x***_*it*_ represents the word vector of the tth word in the ith sentence, ***W***_*e*_*w*_*it*_ represents the word vector of word *w*_*it*_, *R*_*wit*_ represents the word’s comprehensive rumor value, *T* represents the length of the sentence, and ***W***_*e*_ is a 200-dimensional word vector obtained via pre-training with the Word2Vec tool.

*Word sequence encoding*: The forward LSTM layer processes the sequence from left to right by connecting two adjacent units, such as the current unit input *x*_1_ and the hidden state *h*_*t−*1_ of the previous unit input. For a given input sequence *x*_1_, *x*_2_, …, *x*_*it*_, the forward LSTM layer generates an output sequence “$\vec{h}$”. The formula used to calculate the forward LSTM layer is presented in Eq. (4):

4 $$\overrightarrow {{h_{{it}} }} = \overrightarrow {{{LSTM}\left( {x_{{it}} } \right)}}$$

The reverse LSTM layer processes the sequence from right to left by connecting two adjacent units; for example, the hidden state of the input *x*_1_ of the current unit and the input of the next unit *h*_*t*+1_. For a given input sequence *x*_*it*_, …, *x*_2_, *x*_1_, the reverse LSTM layer generates the output sequence “$\overleftarrow {h}$”. The formula used to calculate the reverse LSTM layer is shown in Eq. (5):

5 $$\overleftarrow {{h_{it} }} = \overleftarrow {{{LSTM}\left( {x_{it} } \right)}}$$

The forward and reverse output are combined in Eq. (6):

6 $$\overleftrightarrow {h_{it} } = \overrightarrow {{h_{it} }} \oplus \overleftarrow {{h_{it} }}$$

Here, $\overrightarrow {{h_{it} }}$ represents the forward LSTM layer output value of the tth word in the ith sentence, $\overleftarrow {{h_{it} }}$ represents the output value of the reverse LSTM layer of the tth word in the ith sentence, and $\overleftrightarrow {h_{it} }$ represents the BiLSTM encoding output value of the tth word in the ith sentence.

*Word attention*: The multi-head attention model involves stacking several basic units of scaled dot-product attention. Here the input matrix is Query(***Q***), Key(***K***), Value(***V***), and ${\varvec{Q}},{\varvec{K}},{\varvec{V}} \in {\mathbb{R}}^{n \times d}$, the scaled Dot-Product Attention consists of *h* layers, and the attention calculation of each layer is as shown in Eq. (7):

7 $$\rm{Attention}\left( {\varvec{Q,K,V}} \right) = \rm{softmax}\left( {\frac{{\varvec{QK}^{T} }}{{\sqrt {d_{k} } }}} \right)\varvec{V}$$

Here, *d* is the number of hidden units in the neural network. Because the attention mechanism used by the multi-head attention model is self-attention, the input vector ***Q*** = ***K*** = ***V***. Linear transformation is required for calculation, and the parameters of ***Q***, ***K*** and ***V*** differ each time. When calculating the weights of ***Q*** and all ***K***, the point product similarity function is used, and is scaled through dividing by K dimensions to avoid the problem of an overlarge internal product value. The softmax function is then used to normalize the weights, which are then weighted and summed with the corresponding key values to obtain the attention. The results obtained after h iterations of attention reduction are spliced, after which the values obtained via linear transformation are used as the results of the multi-head attention model. The calculation equation is as shown in (8) and (9):

8 $${\rm head}_{i} = {\rm Attention}\left( {{\varvec{QW}}_{i}^{{\varvec{Q}}} ,{\varvec{KW}}_{i}^{{\varvec{K}}} ,{\varvec{VW}}_{i}^{{\varvec{V}}} } \right)$$

9 $${\rm MultiHead}\left( {{\varvec{Q}},{\varvec{K}},{\varvec{V}}} \right) = {\rm Concat}\left( {{\rm head}_{{1}}, {\rm head}_{{2}}, \ldots, {\rm head}_{{h}} } \right){\varvec{W}}^{o}$$

Here, ${\varvec{W}}^{o}$ represents the weight of linear transformation, while *s* represents the calculated MultiHead (***Q***, ***K***, ***V***) value, which is used to represent more feature information learned from different positions or spaces, as shown in Eq. (10):

10 $$s = {\rm MultiHead}\left( {{\varvec{Q}},{\varvec{K}},{\varvec{V}}} \right)$$

The Max pooling layer is then used for compression change to obtain the most influential sequence $S_{i} ,i \in \left[ {1,L} \right]$, where *L* represents the number of statements.

### Key points of sentences

Sentence key points are obtained from the output of word key points. There are two steps involved, including sentence sequence coding and sentence attention.

*Sentence sequence coding*: Because the semantic features of each sentence affect the credibility of the entire microblog, BiLSTM is used to mine the semantic features between sentences in text. Its calculation equation is shown below in (11):

11 $$\left\{ \begin{gathered} \overrightarrow {{h_{i} }} = \overrightarrow {{{LSTM}\left( {S_{i} } \right)}} \hfill \\ \overleftarrow {{h_{i} }} = \overleftarrow {{{LSTM}\left( {S_{i} } \right)}} \hfill \\ \overleftrightarrow {h_{i} } = \overrightarrow {{h_{i} }} \oplus \overleftarrow {{h_{i} }} \hfill \\ \end{gathered} \right.$$

Here, $\overrightarrow {{h_{i} }}$ represents the output of the ith statement through forward LSTM coding,$\overleftarrow {{h_{i} }}$ indicates the output of the ith statement through reverse LSTM coding, and $\overleftrightarrow {h_{i} }$ represents the output of the ith statement encoded by BiLSTM. The encoded output will then be sent to the sentence attention layer to identify the most important part.

*Sentence attention*: To identify high-impact sentences in the text, each word must be combined with all other words in the sentence weight calculation of the sentence coding sequence using multivalent attention. The characteristic representation *v* is obtained as shown in Eq. (12):

12 $$v = {\rm MultiHead}\left( {{\varvec{Q}}^{\prime } ,{\varvec{K}}^{\prime } ,{\varvec{V}}^{\prime } } \right)$$

Following calculation, the Max pooling layer is used to compress and change, after which the sentence sequence *v*_*i*_ with the most influence is obtained.

### Content credibility evaluation

Once the previous steps are complete, the key point *v*_*i*_ of a microblog represents its semantic features. The microblog content credibility evaluation layer determines the deep-level feature information following multi-layer learning by constructing a true and false binary classification decider for the semantic features of the microblog text, thereby obtaining the final content credibility evaluation result. In this paper, the softmax function is used to construct the credibility classifier, and the calculation equation is as shown in (13):

13 $$p = {\rm softmax}\left( {W_{c} v_{i} + b_{c} } \right)$$

Here, *p* represents the probability of the microblog content being true or false. In this paper, the objective function uses the negative log-likelihood function as the training loss function, the calculation equation for which is as shown in (14):

14 $${\mathcal{L}} = - \sum\limits_{d}^{{}} {\log } \left( {p_{{dz}} } \right)$$

Here, *z* represents the true or false label of the text *d*.

## Experimental results and evaluation

### Experimental environment and related settings

In this paper, the experimental hardware platform is Intel Xeon(2.20 GHz), 12G memory, NVIDIA Tesla P100 16 GB. The experimental software platform is Ubuntu 18.04 operating system and development environment is Python3.6 programming language.

Ten-fold cross validation was used in the experiments^50^, with the average score of ten-fold cross validation being used to indicate the final model performance. The evaluation indexes were accuracy, precision, recall and F1 score. The adjustable parameter settings of CECKP are listed in Table 2.

**Table 2:** Adjustable parameter settings.

Adjustable parameters	Value
Vector embedding dimension	200
Learning_rate	0.001
Optimizer	Adam
Batch_size	64
Dropout	0.3
Number of layers for multi-head attention	8

There are many deep learning-based models at this stage. These novel models have different emphases for different processing objects. When comparing experiments with the model proposed in this paper, a lot of problems may arise. Therefore, it is a good choice to choose baseline models when comparing. To test the performance of the CECKP model, a comparative experiment was conducted with the relevant baseline models, including a classifier method (SVM^51^) and several deep learning methods (CNN^52^, R-CNN^53^, H-BLSTM^34^, Att-BILSTM^54^). The parameter settings of the comparison experimental models are set according to the relevant settings in the reference papers, while ten-fold cross validation is also adopted for the data division method.

### Experimental results and analysis

**Figure 3:**
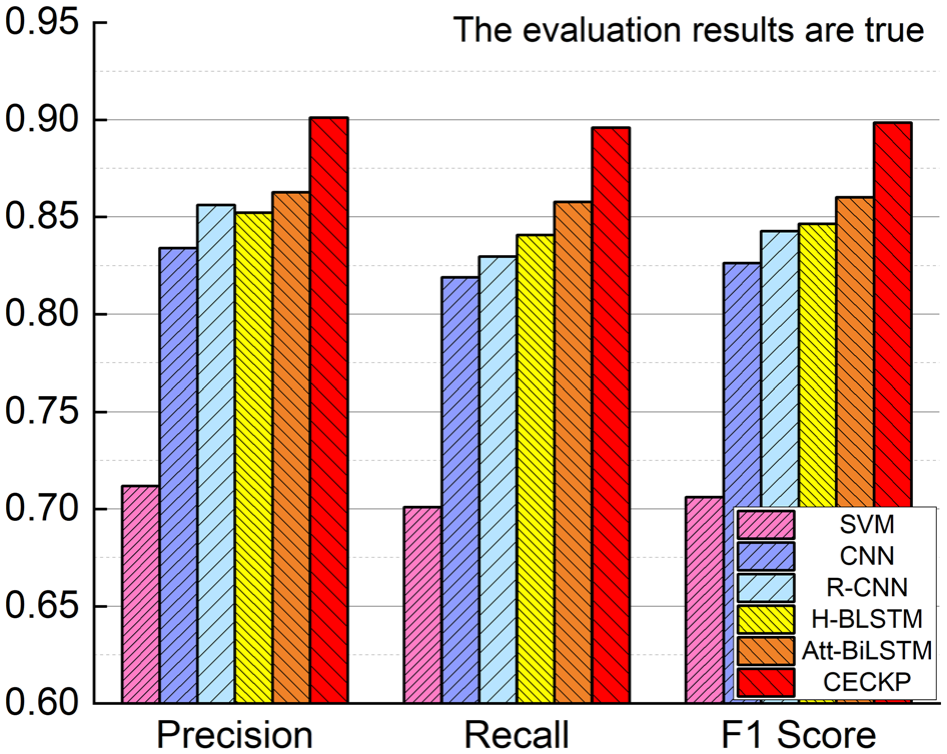
Performance comparison of models on the CECKP-dataset when evaluation results are true.

**Figure 4:**
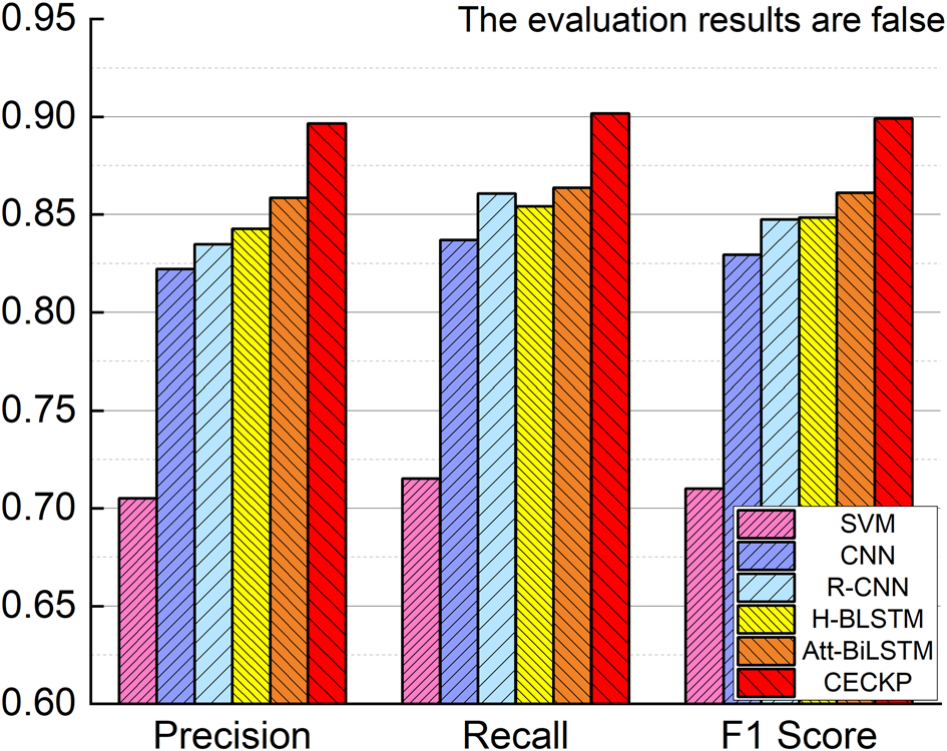
Performance comparison of models on the CECKP-dataset when evaluation results are false.

In this paper, several models are used to verify the validity of the CECKP model. The total accuracy, precision, recall and F1 score for each model in the CECKP-Dataset were obtained and presented in Table 3. To visually illustrate the differences in the obtained values, a bar chart is used to compare the results. A comparison of “true” evaluation results is plotted in Fig. 3, while a comparison of “false” evaluation results is shown in Fig. 4.

**Table 3:** Experimental results for CECKP model and comparison models.

Model	Classification	Accuracy	Precision	Recall	F1 Score
SVM	False	0.7082	0.7052	0.7153	0.7102
	True		0.7119	0.7010	0.7061
CNN	False	0.8280	0.8222	0.8370	0.8295
	True		0.8340	0.8190	0.8264
R-CNN	False	0.8452	0.8348	0.8607	0.8475
	True		0.8562	0.8297	0.8427
H-BLSTM	False	0.8475	0.8428	0.8543	0.8485
	True		0.8523	0.8407	0.8465
Att- BiLSTM	False	0.8607	0.8585	0.8637	0.8611
	True		0.8628	0.8577	0.8602
CECKP	False	0.8988	0.8966	0.9017	0.8991
	True		0.9011	0.8960	0.8985

The CECKP model obtains the key points of words and sentences through multi-part processing of microblogs, composes the important words contained in a sentence into a representation of the sentence, then composes the important sentences in the text into a representation of the text, and subsequently obtains the final key points of the microblog; the obtained key points reflect the semantic features of the microblog text to the greatest extent, and were thus found to yield good evaluation results. Compared with other models, our approach enables more accurate microblog semantics to be obtained, so that good evaluation results can be achieved. Att-BiLSTM can learn top-down and down-top data features, while also adding an attention mechanism to focus the model on more important data, meaning that the overall effect is better. The difference between Att-BiLSTM and the model proposed in this paper lies in the attention mechanism in Att-BiLSTM being calculated only once, while in CECKP, multiple attentions are calculated several times to get the key points of the microblog; this indicates that the attention mechanism plays an important role in content credibility assessment-related research.

### Model simplification test

To further verify the effectiveness of the CECKP model, the following models were constructed: (1) the CECKP-NT model, in which only the multi-head attention model is used, while the collaboration with the rumor word database is not introduced when acquiring the word encoding; (2) the CECKP-NK model, which represents the use of two-way LSTM to encode text content. When this encoding is performed, it is coordinated with the rumor vocabulary, and the multi-head attention model is not used. The experimental results of the model simplification test are presented in Table 4.

**Table 4:** Experimental results of model simplification test.

Model	Classification	Accuracy	Precision	Recall	F1 Score
CECKP-NT	False	0.8752	0.8827	0.8653	0.8739
	True		0.8679	0.8850	0.8764
CECKP-NK	False	0.8683	0.8576	0.8833	0.8703
	True		0.8797	0.8533	0.8633
CECKP	False	0.8988	0.8966	0.9017	0.8991
	True		0.9011	0.8960	0.8985

As can be seen from the above results, the collaborative key points-based method for the credibility evaluation of microblog content is significantly more effective. When the multi-attention model is not used, the key points in the microblog cannot be obtained and the text is not fully mined, resulting in the F1 score for fake microblog detection decreasing by 0.0288. When acquiring the word encoding, there is no coordination of the lexicon, and the semantic enhancement of part of the known rumor words is lost, resulting in a decrease of 0.0252 in the false microblog detection F1 score. According to the experimental results, the acquisition of key points has a slightly greater influence on the credibility evaluation task than the rumor lexicon. However, if the rumor lexicon was to be further expanded in subsequent research, its effect may be improved.

### Visualized analysis

**Figure 5:**
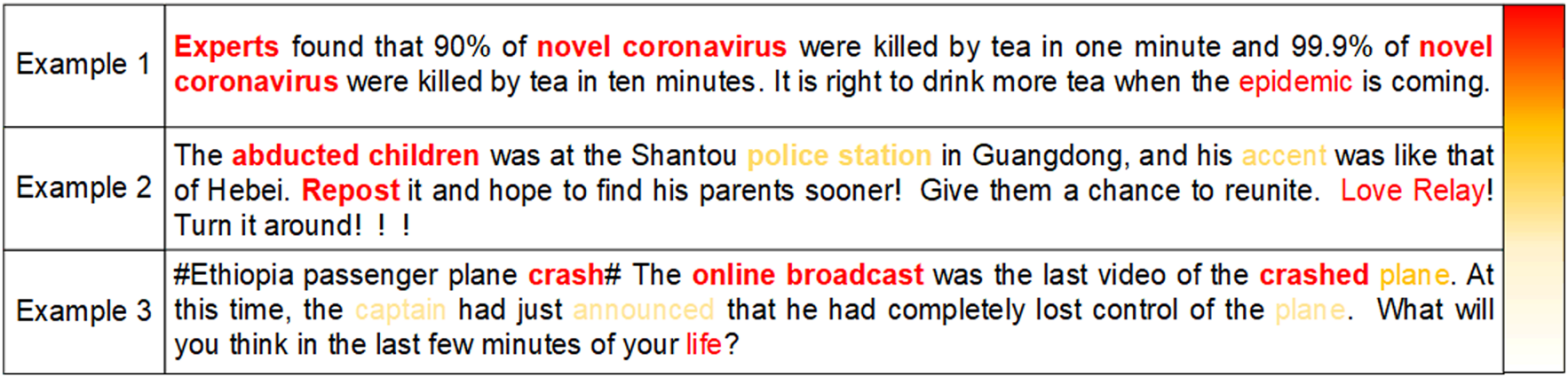
Visual analysis of the weights of key points for rumor words.

To further verify the validity of the CECKP model proposed in this paper in terms of word attention, three fake microblog messages are selected for more in-depth visual analysis. In Fig. 5, the color depth is used to represent the weight of words following key point calculation: the larger the weight, the darker the color. Combine these words as the key points of the text.

It can be seen that the model proposed in this paper is able to select words with a certain comprehensive rumor value, and can also select words that appear repeatedly in the text and have significant meaning; examples include nouns such as experts, novel coronavirus, epidemic, etc. The key points can better represent the text semantics and improve the evaluation effect. However, because these words appear more frequently in both false and true microblogs, it is necessary to get the key points of the full text in conjunction with other words and sentences composed of keywords in the full text, then jointly evaluate the content credibility; otherwise, assessment errors may arise.

### Pre-trained language models analysis

Pre-trained language models were very important in the task of credibility evaluation.We analyzed several commonly used pre-trained language models early in our research, including Word2Vec, BERT, and random embeddings.

Among them, Word2Vec was suitable for non-context-related word vectors. Under this model, similar words will have similar vectors. Bert applied to context-based word vectors, and obtained word vectors for each token based on the input sentence and context. Random embeddings were more advantageous in saving training time and computing resources, but its capabilities were limited.

Bert was better at analyzing sentences with complex structures or ambiguous words in sentences, but it required a long training time, and BERT has a great influence on the distribution of the corpus. When the length of the text we want to represent changes, it will have an impact on model training.

Although Word2Vec cannot obtain the context vector, it can obtain a stable vector training result, and the word vector of the same word can be directly used. The difference is that the word vectors obtained with Bert will change with the context, resulting in a large increase in computational time.

Considering these factors, we choose the Word2Vec model as the pre-trained language model, and the rumor lexicon we build can complement the Word2Vec model to a certain extent.

## Conclusion

To solve the problem of feature selection in content credibility evaluation, this paper proposes a microblog content credibility evaluation model based on collaborative key points (CECKP). In this model, the key points of words and sentences in the text are acquired by means of a multi-attention mechanism, while a rumor lexis is jointly constructed during the acquisition of the key points of words to strengthen the word semantics; subsequently, the credibility of the microblog content is evaluated through the obtained microblog key points. Ten-fold cross validation experiments prove that the proposed model has high accuracy, precision, recall, and F1 score when evaluating the credibility of microblog content. In addition to the text semantic features, microblogs also have many other related features. Currently, multi-modal feature fusion is being increasingly applied in various classification tasks^55,56^. In future work, we will focus on the application of attention mechanisms in multi-modal feature fusion to automatically distinguish various modal features according to weight. A more convenient and efficient assessment of content credibility can be achieved through the use of a relevant attention mechanism.

## Data Availability

The data used to support the findings of this study are available from the corresponding author upon reasonable request.
